# Edible Films from Carrageenan/Orange Essential Oil/Trehalose—Structure, Optical Properties, and Antimicrobial Activity

**DOI:** 10.3390/polym13030332

**Published:** 2021-01-21

**Authors:** Jancikova Simona, Dordevic Dani, Sedlacek Petr, Nejezchlebova Marcela, Treml Jakub, Tremlova Bohuslava

**Affiliations:** 1 Department of Plant Origin Food Sciences, Faculty of Veterinary Hygiene and Ecology, University of Veterinary and Pharmaceutical Sciences Brno, Palackeho tr. 1946/1, 612 42 Brno, Czech Republic; dordevicd@vfu.cz (D.D.); tremlovab@vfu.cz (T.B.); 2 Faculty of Chemistry, Brno University of Technology, Purkynova 118, 612 00 Brno, Czech Republic; sedlacek-p@fch.vut.cz; 3 Department of Molecular Pharmacy, Faculty of Pharmacy, Masaryk University, Palackeho tr. 1946/1, 612 00 Brno, Czech Republic; nejezchlebovam@pharm.muni.cz (N.M.); tremlj@pharm.muni.cz (T.J.)

**Keywords:** trehalose, orange essential oil, antimicrobial activity, transparency value, edible packaging

## Abstract

The research aim was to use orange essential oil and trehalose in a carrageenan matrix to form edible packaging. The edible packaging experimentally produced by casting from an aqueous solution were evaluated by the following analysis: UV-Vis spectrum, transparency value, transmittance, attenuated total reflectance Fourier-Transform spectroscopy (FTIR), scanning electron microscopy (SEM) and antimicrobial activity. The obtained results showed that the combination of orange essential oil with trehalose decreases the transmittance value in the UV and Vis regions (up to 0.14% ± 0.02% at 356 nm), meaning that produced films can act as a UV protector. Most produced films in the research were resistant to Gram-positive bacteria (*Staphylococcus aureus* subsp. *aureus*), though most films did not show antibacterial properties against Gram-negative bacteria and yeasts. FTIR and SEM confirmed that both the amount of carrageenan used and the combination with orange essential oil influenced the compatibility of trehalose with the film matrix. The research showed how different combinations of trehalose, orange essential oils and carrageenan can affect edible film properties. These changes represent important information for further research and the possible practical application of these edible matrices.

## 1. Introduction

The main functions of food packaging are protection against the outer environment, dust, microorganisms, pests and radiation too [1]. For example, UV radiation can cause the oxidation of lipid compounds, but there can also be an issue with the discoloration of food that makes food products less attractive for consumers [2,3]. Many different compounds can be used to improve these films’ properties: tannic acid with modified microfibrillated cellulose films has UV blocking ability [4], and the addition of zein nanoparticles and olive oil in the starch–glycerol matrix decreases transmittance and improves the UV barrier [5]. The important advantage of edible packaging is that it is produced from natural materials, and accordingly, it can help to reduce synthetic waste. Additionally, edible packaging can be also consumed together with the packaged/wrapped foodstuff [6].

Incorporation of trehalose into food packaging is suggested for diverse purposes. It helps to preserve the aroma and color of dried fruits and improve weight loss and color of packaged vegetables, but no differences in firmness were found between coated and uncoated samples [7,8]. Trehalose addition to food packaging material can also prevent a reduction in organic acid content during storage [9]. Another biological advantage of trehalose is its capability to improve plant cells’ viability after freezing and subsequent thawing [10]. Furthermore, the results of the previous works indicated that trehalose also possesses UV barrier properties [11].

Essential oils are also proposed as promising additives for food packaging because they represent a well-functioning nature-based alternative to synthetic antioxidant compounds such as BHA (butylhydroxyanisole) or BHT (butylhydroxytoluene). Nevertheless, the direct use of essential oils in foodstuffs is limited because the concentration of active compounds in them is very high. The intense aroma of essential oils, noticeable even at very low concentrations, can affect sensory properties negatively [12]. Moreover, the inherent antimicrobial activity of essential oils is partially reduced in vitro since the compounds interact with food matrices and decrease antimicrobial activity. However, the use of essential oils in food packaging can be highly recommended because it can work as the active packaging; some compounds can gradually migrate to foodstuff and improve their properties (shelf life, etc.). Certainly, the release rate is affected by the package material structure and composition. The compounds from essential oils incorporated into edible packaging can slowly be released into the food [13,14,15]. As with trehalose, orange essential oil possesses UV protective properties too [16]. Orange essential oil is produced from fruits of *Citrus aurantium* L., Rutaceae containing mainly D-limonene and myrcene [17].

Among the sensory properties of food packaging, transparency is an outstandingly important parameter since the consumers may buy food based on the transparency of packaging material. According to literature data, transparent materials are more attractive for consumers since they are more visually informed about the food before purchase and foodstuffs can look appetizing packed with this material type [18].

The microbial food contamination poses a serious problem for producers, shortens on-shelf time, and may lead to life-threatening infections in humans. There are many microorganisms that can contaminate certain food, and for that reason, we chose three model microorganisms: Gram-positive *Staphylococcus aureus*, subsp. *aureus*; Gram-negative *Escherichia coli*; and a yeast *Candida albicans* [19].

The addition of essential oils in edible matrices was studied in some previous research, but in many of them, the main characteristics were physical, mechanical properties and antimicrobial activity [20,21,22]. Only in a few of them the UV-Vis properties were studied. The importance of UV-Vis properties can be seen through the fact that these properties explain the capability of biodegradable packaging to protect food against radiation that can lead to food spoilage [2,3]. It should be also stressed that there have not been publications dealing with the preparation of edible films that included orange essential oils and trehalose in one film matrix.

Our study focused on the experimental production of edible films that consisted of carrageenan, trehalose and orange essential oil. The study aimed to monitor the influence of these compounds on the UV-Vis properties and their mutual interactions in the experimentally produced edible films.

## 2. Materials and Methods 

### 2.1. Material and Preparation of Edible Films

The κ-carrageenan, Tween 20 (T20) and Tween 80 (T80) were obtained from Sigma Aldrich (St. Louis, MO, USA). The orange essential oil (OEO) was purchased from the local market (dm-drogerie markt GmbH&Co KG, Brno, Czech Republic). Trehalose (Tr) was obtained from Toppotraviny.cz s.r.o. (Prague, Czech Republic). 

Films were prepared by aqueous solution casting method as follows: The respective weight of carrageenan (0.5 or 0.3 g) was dispersed in 43.65 mL of distilled water. A corresponding amount of trehalose was added together with carrageenan to set the final concentration of trehalose in solution at 0%, 0.5%, 1% and 3% (*w*/*v*), respectively. The complete dissolution was assured via mixing the dispersion on a magnetic stirrer (50 °C, 350 rpm) for 10 min. For the samples containing essential oil, 0.45 mL of orange essential oil was added in the next step and a homogeneous emulsion was achieved by another 5 min of stirring (50 °C, 350 rpm). In the resultant dispersion, 0.25 mL of glycerol was dissolved and, in the case of emulsions containing OEO, 1.3 mL of Tween 20 or Tween 80, was added 5 min after the glycerol addition, and the final mixture was stirred for 7 more minutes at 850 rpm. The films were prepared by casting the final dispersion on the Petri dishes with a diameter of 9 cm, and the films were dried for 24 h. The tested compositions of the edible films are described in Table 1.

### 2.2. UV-Vis Spectra and Transparency Value

The UV-Vis spectra were recorded by a spectrophotometer CE7210 (DIET-QUEST, Cambridge, UK) at wavelengths from 200 to 600 nm. The transmittance values (%) were calculated at wavelengths 356 and 600 nm. The transparency values were then calculated according to Han and Floros [23] as follows:

Transparency value = logT_600_/x (1)

where T_600_ is the transmittance (%) at 600 nm and x is the thickness of film samples (mm).

### 2.3. Attenuated Total Reflectance Fourier-Transform Spectroscopy

Fourier transform infrared spectra of the prepared films were measured with an iS50 Fourier-Transform spectrometer (FTIR) (Thermo Scientific, Waltham, MA, USA). All measurements were taken from a surface of a film at ambient temperature (in an air-conditioned room) with the built-in single-reflection diamond attenuated total reflectance (ATR) crystal. An individual absorption spectrum was collected as an average of 16 scans with a resolution of 4 cm^−1^ (data spacing 0.5 cm^−1^). Each film was analyzed at 5 randomly distributed spots on its surface; FTIR spectra provided in respective figures in this paper represent an average of all the spectra collected for an individual sample.

### 2.4. Scanning Electron Microscopy

Micrographs of all prepared films were recorded using a Zeiss EVO LS-10 scanning electron microscope (SEM) (Carl Zeiss Ltd., Cambridge, UK). Before the SEM analysis, a small cut-off of each sample (ca 10 × 10 mm^2^) was stuck on a carbon tape and sputter-coated with gold.

### 2.5. Antimicrobial Activity

The edible packaging material was exposed to UV light (wavelength 260 nm) for physical disinfection before the testing. Subsequently, discs with a diameter of 5 mm were cut from the material in an aseptic environment. A modified disk diffusion method according to the European Committee on Antimicrobial Susceptibility Testing (EUCAST) was used to determine the antimicrobial resistance of edible packaging.

The solid medium according to Mueller and Hinton (MUELLER-HINTON broth, Agar for microbiology, Sigma-Aldrich) was used for culturing *Staphylococcus aureus* subsp. *aureus* CCM 7110 and *Escherichia coli* CCM 3954 with edible coatings, and solid medium Malt (Malt Extract Broth, Agar for microbiology, Sigma-Aldrich) was used for culturing of *Candida albicans* CCM 8261 with edible coatings. The inoculum concentration was adjusted to approximately 1–2 × 10^8^ CFU/mL, corresponding to 0.5 degrees of McFarland turbidity standard. An amount of 1 mL of inoculum was spread on the surface of the agar and, after drying, 6 cut-off discs from edible packaging material were placed on the agar. The inoculated plates were incubated for 18 h at 35–37 °C. The evaluation of antimicrobial activity was made by observation with the naked eye. To describe the results, we developed an evaluation scale from 1 to 5 (1 = disc completely clean, 5 = disc completely overgrown). According to the value, the result designated in Table 4 is either “antimicrobial activity” (+; values 1 and 2) or “no activity” (-; values 3, 4 and 5).

Depending on the medium, microorganisms and type of edible packaging, in some cases, the packaging material melted during cultivation at 35–37 °C.

Reference strains of *Staphylococcus aureus* subsp. *aureus* CCM 7110, *Escherichia coli* CCM 3954 and *Candida albicans* CCM 8261 were obtained from the Czech Collection of Microorganisms of the Department of Experimental Biology, Faculty of Science, Masaryk University.

### 2.6. Statistical Analysis

Statistical analysis was performed by one-way ANOVA analysis of variance, when statistical significance at *p* < 0.05 was evaluated. A parametric Tukey post-hoc test and nonparametric Games–Howel post hoc test were used for finding differences within groups. Principal component analysis (PCA) was used for the determination of overall differences among samples. SPSS 20 statistical software (IBM Corporation) was used for all statistical evaluation.

## 3. Results and Discussion

### 3.1. UV-Vis Spectra

**Figure 1:**
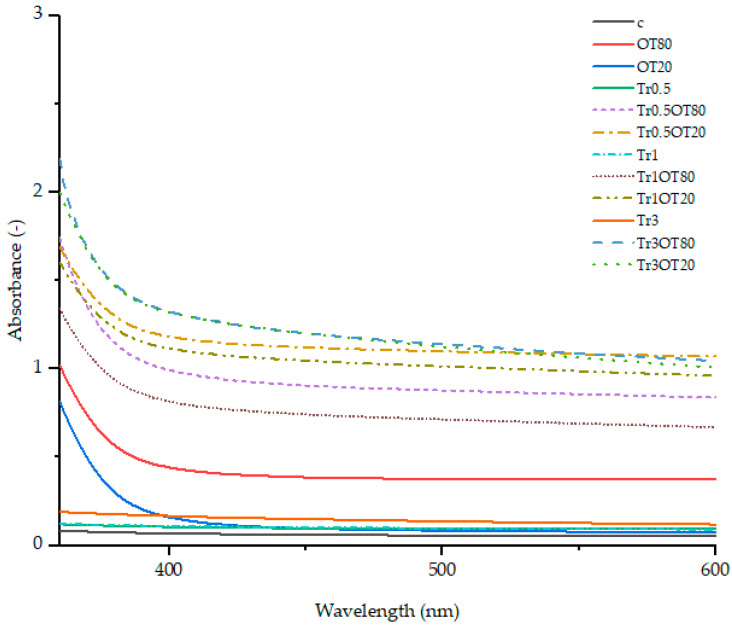
UV-Vis spectra of films with the addition of 0.5 g κ-carrageenan.

**Figure 2:**
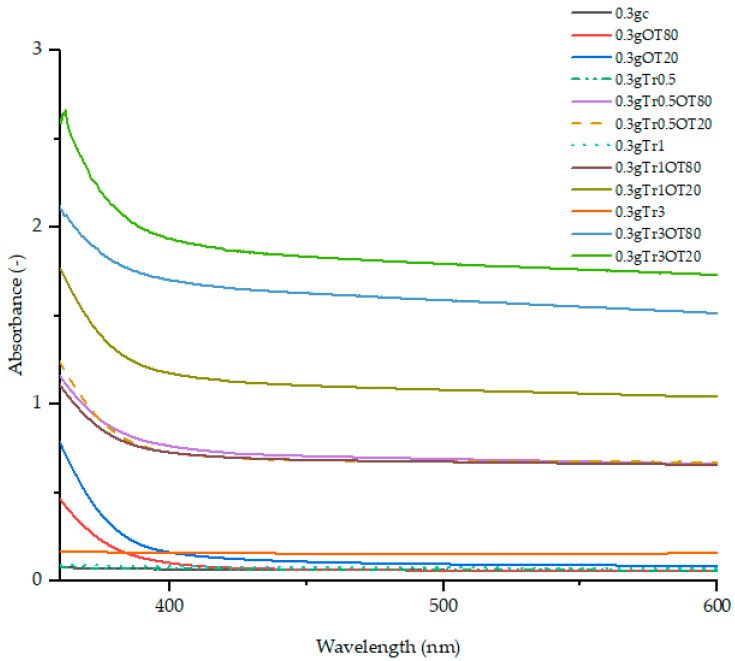
UV-Vis spectra of films with the addition of 0.3 g κ-carrageenan.

The results of UV-Vis spectroscopy analysis are shown in Figure 1 (for film samples with 0.5 g of κ-carrageenan) and in Figure 2 (for film samples with 0.3 g of κ-carrageenan). In general, requirements of the spectral properties of packaging materials in UV-VIS regions are rather complex and often contradictory. On the one hand, low transmittance of UV radiation is highly regarded as far as it increases the lifespan of the packaged foodstuff. High transparency of the packaging is required in the visible region since it provides a consumer with a visual control of the commodity’s condition. 

The figures show absorbance spectra in the spectral range from 356 to 600 nm; bellow 356 nm, the absorbance value exceeded the sensitivity limit of the spectrophotometer as it was rising above three. Based on this high absorbance in the UV region, the films have good potential barrier properties against UV radiation [24,25].

The UV-Vis spectra of samples with 0.5 g of κ-carrageenan were shifted to the higher values of absorbance when the essential oil, trehalose and Tween 80 or Tween 20 were added. The lowest absorbance was found in the control sample; this finding is in accordance with the previous experiment [26]. When only essential oil with emulsifier Tween 20 was added, the absorbance increased in the UV region, but not in the visible region. On the other hand, when Tween 80 was added, the absorbance increased in UV as well as in the VIS region. The combination of essential oil with trehalose resulted in higher absorbance (positive correlation with trehalose). The highest results of absorbance were found in the samples Tr3OT80 and Tr3OT20 (the samples with the highest concentration of trehalose: 3%). In the previous research conducted by Liu et al. [11], it was found that trehalose could work as a protective agent against UV radiation.

In Figure 2, the lowest UV-VIS spectra were found for 0.3gc (the line hidden behind the line for 0.3gTr0.5 and 0.3gTr1). The samples without the addition of orange essential oil had the lowest absorbance. The addition of orange essential oil increased the absorbance, and the combination with orange essential oil and trehalose resulted in the highest absorbance (0.3gTr3OT20 and 0.3gTr3OT80). A higher absorbance of films with OEO can be caused by the presence of lipid droplets in films that have a different refractive index, inducing light scattering [27]. The study emphasized that the combination of OEO and trehalose multiplies protection against UV radiation, meaning that there is a synergistic effect between these two substances. The combination of oil and sugar added to the matrix of edible film causes the formation of oil droplets; the increasing concentration of sugar causes less breaking of forming droplets, and oil droplets occur in a larger size [28].

### 3.2. Transmittance

The results for transmittance are shown in Table 2. It has to be stressed that when the Tween 80 or Tween 20 and orange essential oil were added, the transmittance in the UV region at 356 nm rapidly decreased; the same happened at 600 nm. Haghighi et al. [29] found similar results when the combination of polymer and essential oils was studied. In our samples, the lowest transmittance as well as the best UV barrier properties were found in samples with the addition of 3% trehalose and orange essential oil. The addition of higher amounts of trehalose resulted in a transmittance decrease. Statistically significant differences (*p* < 0.05) were not found between all samples with the addition of essential oil in T356. However, statistically significant differences (*p* < 0.05) were found between samples without orange essential oil and with essential oil, so the addition of orange essential oil and Tween 80 or Tween 20 had a higher impact on the transmittance of prepared films. The transmittance, the same as absorbance, was affected by the presence of oil droplets in edible films [27,28]. The addition of trehalose also had an impact; statistically significant (*p* < 0.05) differences were found between c, Tr0.5 and Tr1. Sample Tr3 was statistically similar to Tr0.5 and Tr1. Hence, it seems that the effect of trehalose on the transmittance properties of the films is plateau-like, i.e., increasing the concentration of trehalose above a certain limit does not result in a further improvement of the optical properties. Transmittance at 600 nm for samples with 0.5 g of κ-carrageenan was affected by the addition of essential oil, where c showed a statistically significant difference (*p* < 0.05) in comparison with OT80 and OT20, but the more pronounced decline in transmittance at 600 nm was found in samples with a combination of trehalose and essential oil. The transmittance of the control sample was the highest, meaning that κ-carrageenan does not possess the same large barrier properties in the UV region as in the Vis region [30]. This is caused by the presence of terpenes in essential oils, since they work as UV protectors [31].

The UV barrier properties are very important for the food packaging industry; common examples are oxidation of lipids and discoloration of food [2,3]. The most affected are unsaturated fatty acids and their esters; the products of photooxidation are hydroperoxides. Hydroperoxides can decompose and produce radicals that induce oxidation of other fatty acids [32]. Other compounds, which are affected by UV, are vitamins and carotenes [33]. Vitamin A and β-carotene are the most affected by wavelengths below 465 nm. β-carotene is more reactive to oxidation than vitamin A acetate; this is explained by the presence of six extra double bonds, in the β-carotene structure [34]. Rincón, et al. [35] also confirmed a decrease in transmittance with the addition of essential oil. Again, the combination of trehalose and OEO provides the best transmittance results.

The protection against UV radiation is not important only due to the protection of packed foodstuffs but also for the protection of the packaging material, because UV radiation can cause the degradation of the polymer material [36,37]. During degradation, the polymer starts to break the polymer chain, free radicals are aroused and the molecular weight is reduced, leading to deterioration of mechanical properties and material destruction [38].

### 3.3. Transparency Value

The transparency values are summarized in Table 3. It can be seen that the addition of orange essential oil and trehalose had a statistically significant impact (*p* < 0.05) on this property. Higher trehalose addition resulted in lower transparency value. The interpretation of transparency value is as follows: the lower the transparency value is, the higher the observed opacity of the films is [39].

**Figure 3:**
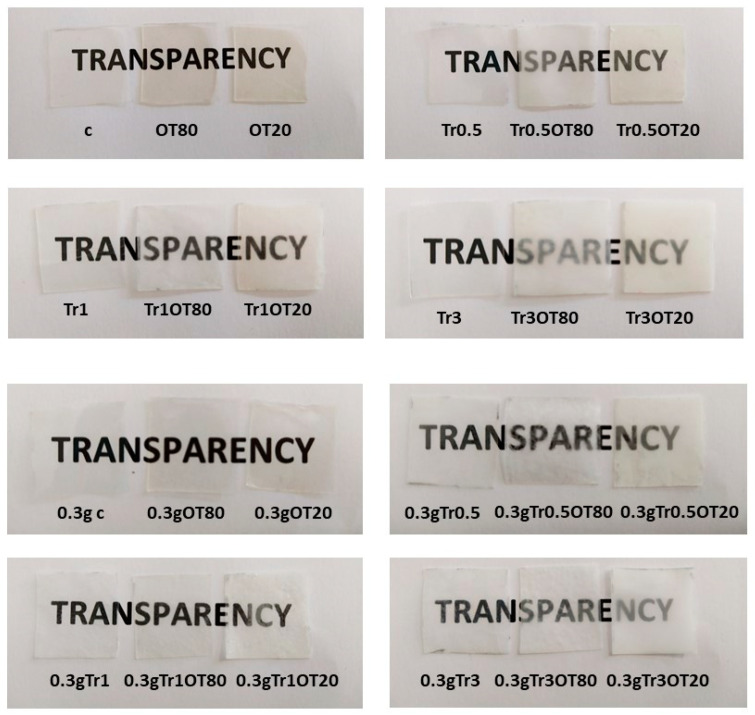
Transparency appearance of edible films.

Transparency value can be compared to the real appearance of prepared films in Figure 3. From the data and pictures in Table 3 and Figure 3, respectively, it is apparent that the addition of orange essential oil highly affected the transparency of films, but it has to be stressed, subjectively, in Figure 3 that the films were less transparent with higher concentrations of trehalose. Nevertheless, the combination of trehalose and orange essential oil affected the transparency too, the same as the addition of orange essential oil without trehalose. Šuput et al. [40] and Shojaee-Aliabadi et al. [41] studied the addition of different essential oils in starch and carrageenan films, and found similar results to those that can be caused by the presence of phenolic compounds in essential oils, which might absorb light at low wavelengths. In our research, the combination of trehalose and OEO had a statistically significant impact (*p* < 0.05) on the transparency value, compared to the samples without trehalose and OEO.

The transparency of films is an important part of marketing, because transparent packaging materials are usually more attractive for consumers, since they are more visually informed about the food before purchase, and foodstuffs can look appetizing packed with this material type [18]. The transparency values showed similar results to transmittance, so the less transparent films can be used as food protectants against the light and prevent lipid oxidation [42].

### 3.4. Scanning Electron Microscopy

**Figure 4:**
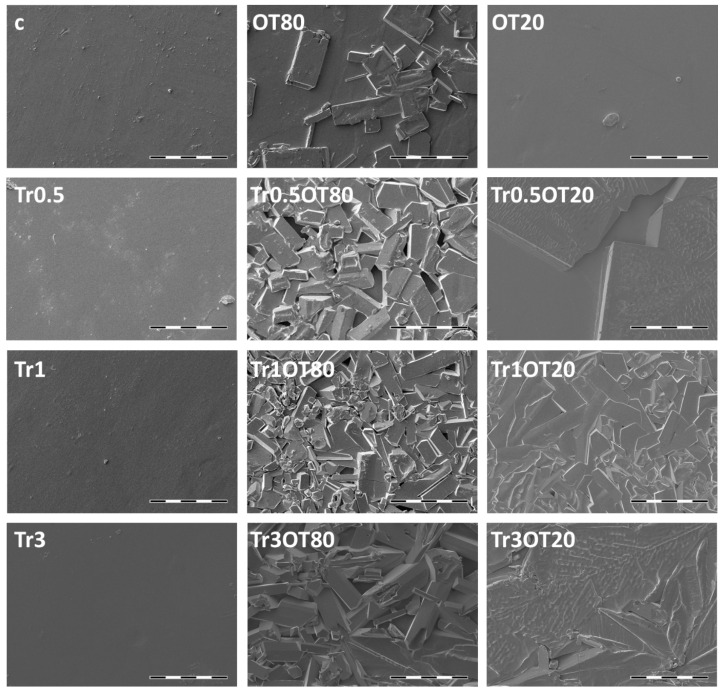
The SEM micrographs of films that consisted of 0.5 g of κ-carrageenan (voltage = 5 kV, current = 60 pA, scale bar = 100 µm).

**Figure 5:**
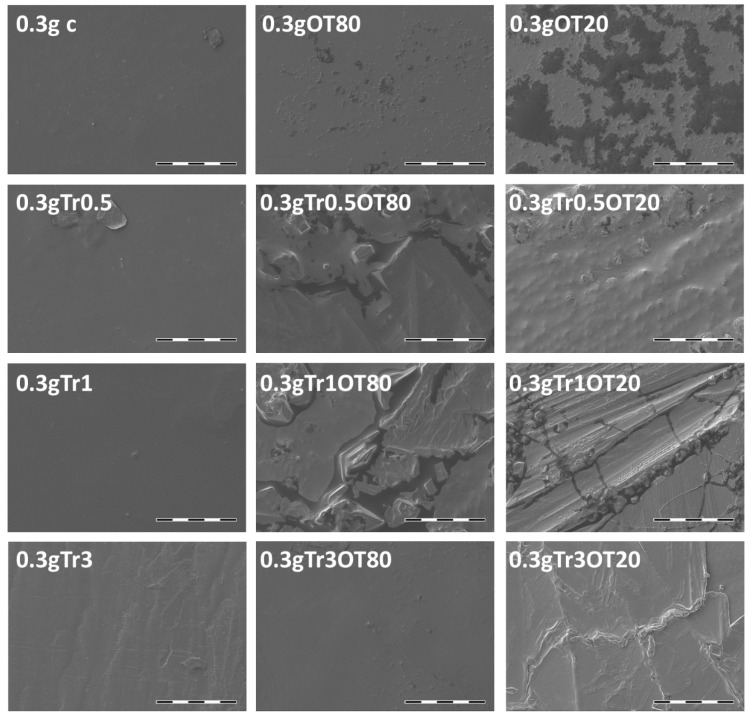
The SEM micrographs of films that consisted of 0.3 g of κ-carrageenan (voltage = 5 kV, current = 60 pA, scale bar = 100 µm).

SEM analysis is commonly used for the description of packaging surfaces, describing their homogeneity and integrity [43]. Results of the SEM analysis of the prepared edible films are shown in Figure 4 and Figure 5 and in also more detail in the Supplementary Materials (Figures S1 and S2). It can be seen that the control samples with both contents of carrageenan (samples c and 0.3g c) show smooth homogeneous surfaces indicating an effective plasticization of the carrageenan by glycerol. Among the other compositions of the films, it can be seen that the films with the lower content of carrageenan (0.3 g) in general maintain the integrity and homogeneity of the surface even after the addition of other components, while films with the higher content of carrageenan (0.5 g) are more prone to a surface heterogeneity. At this content of carrageenan, a moderate presence of separated agglomerates and/or crystals on the surface of the films was found for the OT80, and a significant surface heterogeneity was determined in all samples that contained a combination of essential oil and trehalose. At the same time, from the comparison of the topography of the essential oil-containing films, it is obvious that the use of Tween 20 as the emulsifier results in less heterogeneous topography.

Furthermore, it can be seen that the extent of surface heterogeneity is inversely proportional to the film transparency, which confirms that the number and size of the heterogeneities (crystals, agglomerates) affect the amount of light scattered during the passage through a film. Therefore, from the perspective of integrity and homogeneity of the films, and the resulting optical properties, preparation of the films with the lower content of carrageenan seems to be more appropriate.

### 3.5. Fourier-Transform Infrared Spectroscopy

**Figure 6:**
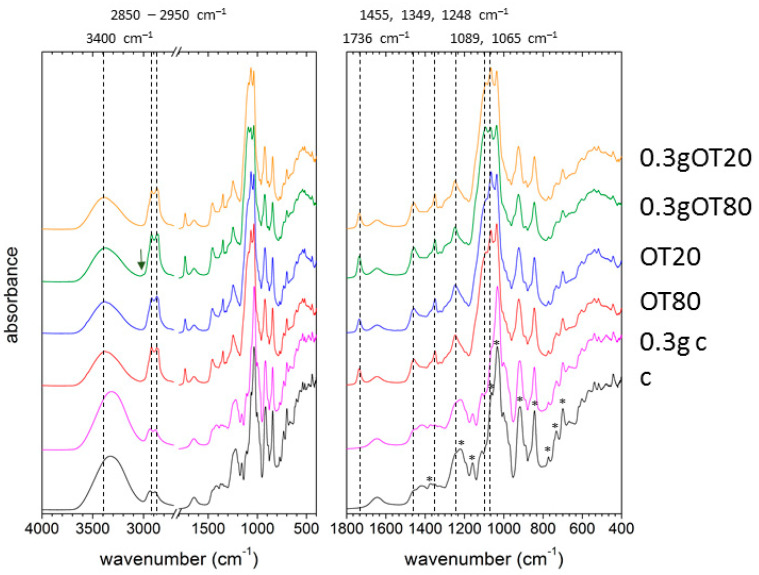
Attenuated total reflectance (ATR) Fourier-Transform spectroscopy (FTIR) spectra of the films prepared without the addition of trehalose.

FTIR analysis was also involved in the study to provide a deeper view into the chemical structure of the prepared films. The attenuated total reflectance (ATR) technique was used to focus the analysis primarily on the surface of the prepared films. FTIR spectra of the films prepared with the absence of trehalose are shown in Figure 6. It can be seen that the presence of the main structural components is reflected in the measured spectra. The spectrum of the control sample (sample c) combines characteristic vibrations of the main film-forming component (carrageenan) and the plasticizer (glycerol). The wide absorption band centered at 3300–3400 cm^−1^ is attributed to stretching vibration of O–H bonds which are found in both the film components as well as in the residual moisture contained in the film. The presence of the free water molecules (i.e., those not bound in the form of a crystalline hydrate) is further confirmed by an absorption band at 1640 cm^−1^ (water molecule bending) and by a characteristic baseline deformation at the lowest measured frequencies. Aside from the contribution to –OH stretching above 3000 cm^−1^, other characteristic vibrations of carrageenan can easily be recognized over the whole analyzed spectral range (the specific vibrations that were assigned according to the literature [44] are marked with an asterisk in Figure 6). First, asymmetric and symmetric stretching vibrations of C–H bonds in carrageenan methylene groups are located at 2940 and 2890 cm^−1^, respectively. Second, the characteristic vibration pattern of oxygen-containing groups in carrageenan can be found in the region from 900 to 1200 cm^−1^, namely, at 920 cm^−1^ (C–O stretching in 3,6-anhydro-d-galactose), 1035 and 1063 cm^−1^ (C–O and C–OH modes and glycosidic linkage) and 1159 cm^−1^ (C–O–C asymmetric stretching). Sulfate-associated vibrations of carrageenan can also be recognized in the spectrum mainly at 843 (d-galactose-4-sulphate), 1225 (sulfate ester asymmetric stretching) and 1375 cm^−1^ (sulfate stretching). Finally, skeleton bending of pyranose is manifested by the specific fingerprint pattern with the sharp peaks at 700, 733 and 772 cm^−1^. Unlike the well-distinguished spectral features of carrageenan, characteristic C–C and C–O vibration bands of glycerol, normally occurring in the range 850–1100 cm^−1^, are not easily identified in the spectra as they are overlapped by the carrageenan signal even at its lower content in the film (see the indifferent spectra of samples c and 0.3g c in Figure 6).

**Figure 7:**
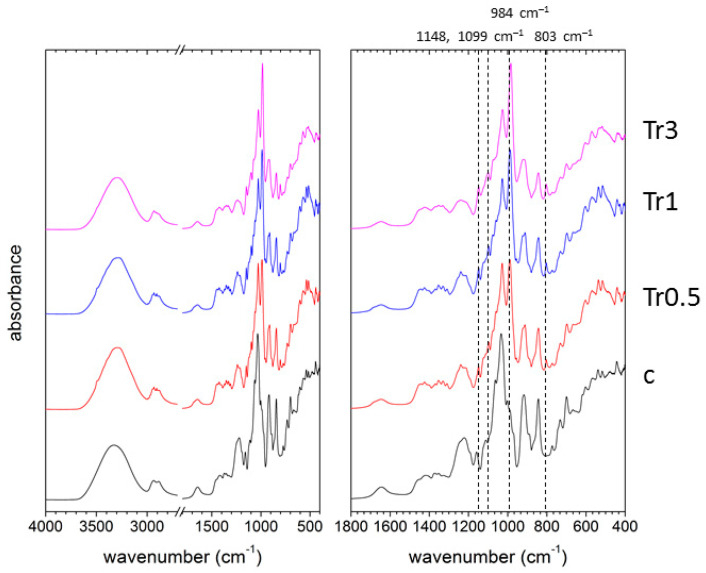
ATR FTIR spectra of the trehalose-containing carrageenan films without the addition of essential oil (0.5 g of carrageenan).

As expected, FTIR spectra of the films containing the essential oil are well-distinguished from the spectra of the corresponding control samples (see Figure 6). Nevertheless, the main differences between the spectra with and without the essential oil, respectively, can be assigned to the presence of emulsifier (Tween 80 and Tween 20, respectively). First, higher intensity of C–H stretching in the range from 2800 to 3000 cm^−1^ can be attributed to the hydrocarbon chains in the surfactants, whereby the peak frequencies of the asymmetric and symmetric vibrations are slightly red-shifted as compared to the controlled sample (to 2920 and 2860 cm^−1^). Furthermore, the spectral region that corresponds to the vibrations of oxygen-containing groups is also significantly altered. Carbonyl stretching in the ester group is manifested by the sharp peak at 1736 cm^−1^, while the presence of ether linkages increases the absorption at 1065 and 1089 cm^−1^, and the –OH bending intensity increases the absorption at 1455 cm^−1^. Furthermore, C-H bending in CH_2_ and CH_3_ is manifested by absorptions at 1349 and 1248 cm^−1^. Both the ether and the methylene moieties appear also in the structure of essential oils; therefore, it is likely that the essential oil incorporated in the films contributes to some of the spectral features assigned to the presence of emulsifier. In general, no significant spectral differences were found between the films prepared with the use of different emulsifiers (Tween 80 and Tween 20, respectively). Among the spectral features related to the presence of the C=C bond in the Tween 80, only the very weak C-H stretching vibration (at 3010 cm^−1^) can be observed in a spectrum of 0.3gOT80 (marked with arrow ion Figure 7), while it is overlapped with the wide and intense –OH stretching band for the sample with a higher content of carrageenan (OT80).

The most interesting IR spectral features were revealed for the films containing trehalose. It is well known that trehalose exhibits polymorphism, i.e., depending on the thermodynamic conditions, it can adopt different crystalline forms aside from the form of amorphous solid [45], whereby each of the adopted forms exhibits specific spectral features in FTIR [46]. The actual adopted form of trehalose is crucial from the perspective of its performance as an active ingredient of the edible films, because it will affect such essential physicochemical parameters as the rate and heat of dissolution in water [47]. Furthermore, it has also been suggested that different crystalline forms may differently contribute to the bioprotective function of trehalose [46].

**Figure 8:**
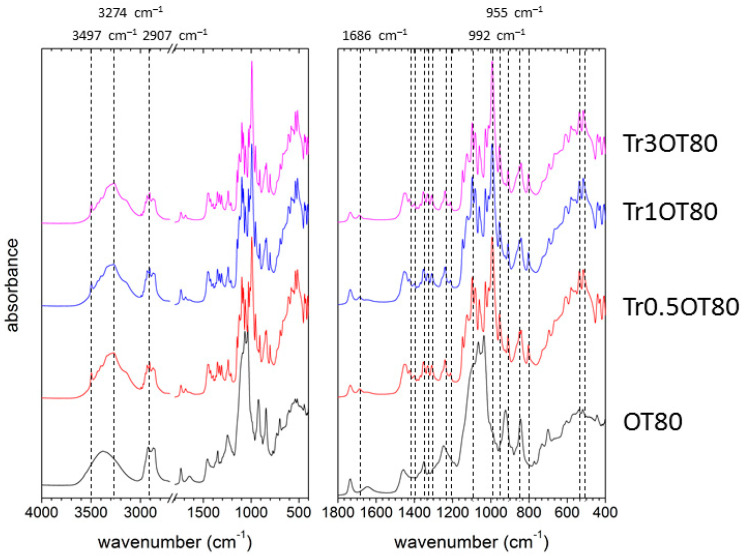
ATR FTIR spectra of the trehalose-containing carrageenan films with the addition of essential oil (0.5 g of carrageenan, Tween 80).

The main vibration bands ascribed to a trehalose presence are those corresponding to C–O–C stretching of the glycosidic bond. These bands occur in the range 900–1200 cm^−1^ depending on the symmetry of the molecule. Therefore, the FTIR pattern of trehalose in this spectral region can be used as a marker of the trehalose conformation [46,48]. Figure 7 shows FTIR spectra of the trehalose-containing films with a higher content of carrageenan (0.5 g) and with the absence of essential oil, while Figure 8 shows spectra of the films also containing essential oil and emulsifier (Tween 80). The difference between the trehalose manifestation in both spectra is obvious. 

While the trehalose signature in spectra shown in Figure 7 is limited to several bands (the most important ones are marked in the figure), significantly more peaks appear in the spectra shown in Figure 8 as a result of the trehalose presence. Comparing the following spectral features with the literature [46], we suggest that the spectral signature of agarose shown in Figure 7 may be attributed to the prevailing content of amorphous trehalose, while that presented in Figure 8 corresponds to the majority of crystalline trehalose dihydrate: (a) In the samples without essential oil, the most intensive vibration band occurs at 984 cm^−1^, while the band is red-shifted to about 992 cm^−1^ for the films with essential oil. (b) Symmetric stretching vibration of the glycosidic bond at 955 cm^−1^ occurs specifically in spectra of the dihydrated crystalline form. (c) Water molecules trapped in the crystalline dihydrate exhibit specific stretching (around 3500 cm^−1^) and bending (around 1680 cm^−1^) frequencies. For the essential oil-containing films with the same concentration of carrageenan but with Tween 20 as the surfactant, similar qualitative signs of the presence of trehalose dihydrate were found (see Figure S3 in the Supplementary Materials). Altogether, these results indicate that trehalose in the films with the higher content of carrageenan is less compatible with the film matrix (i.e., less content of trehalose is dispersed in the film in amorphous form) when the surfactant and the essential oil is added. This also suggests that the surface heterogeneity for the essential oil-containing films as revealed by SEM is caused by crystals of trehalose dihydrate excluded from the film matrix. 

For the films with the lower content of carrageenan (0.3 g), the presence of the essential oil exhibited a less pronounced impact on the trehalose crystallinity (see Figures S5 and S6 in the Supplementary Materials). Except for sample 0.3gTr3OT20, trehalose in the films with the presence of the essential shows predominantly spectral signs of the amorphous form. On the other hand, higher contents of trehalose in the films without OEO bring about higher signs of the dihydrate presence as compared to corresponding films prepared from the higher content of carrageenan. It is likely that while in the case of 0.5g carrageenan films, the trehalose dihydrate crystals excluded on the surface of the films are the main contributors to the collected spectra, in the case of smooth homogeneous films prepared from 0.3 g carrageenan, the FTIR spectra better represent the true distribution of trehalose conformers inside the film matrix. 

**Figure 9:**
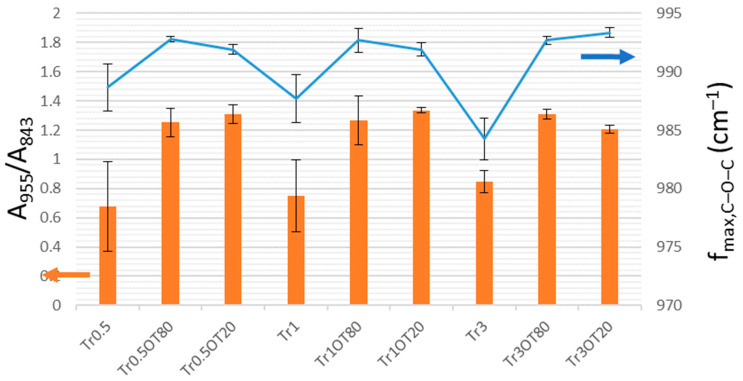
Quantitative crystallinity markers determined from ATR FTIR spectra of the trehalose-containing carrageenan films (0.5 g of carrageenan). A955/A843 represents absorbance ratio at 955 and 843 cm–1; fmax, C–O–C represents the frequency of the maxima for the glycosidic linkage stretching in trehalose.

**Figure 10:**
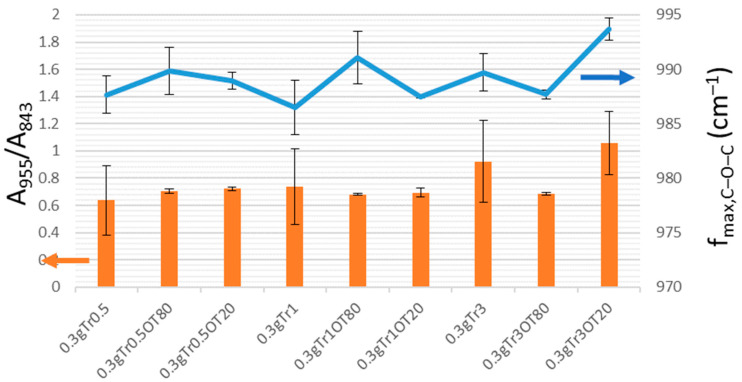
Quantitative crystallinity markers determined from ATR FTIR spectra of the trehalose-containing carrageenan films (0.3 g of carrageenan).

To provide a semi-quantitative evaluation of the relative content of individual trehalose forms from the collected FTIR spectra, we calculated two spectral parameters which should positively correlate with the content of trehalose dihydrate—the absorbance ratio at 955 (trehalose dihydrate) and 843 cm^−1^ (carrageenan) and the frequency of the antisymmetric stretching of the glycosidic bond. The results are shown in Figure 9 and Figure 10. It can be seen that both parameters confirm two main conclusions of the qualitative evaluation of the FTIR spectra, i.e., that in the case of 0.5 g carrageenan films, the presence of essential oil and emulsifier induces exclusion of trehalose dihydrate crystals on the film surface, while the 0.3 g carrageenan films, in general, show higher compatibility of trehalose with the film matrix, hence reducing the relative content of crystalline trehalose dihydrate. The presence of both forms of trehalose can be considered advantageous from different points of view. Maintaining trehalose in the amorphous form dispersed inside the film matrix will prevent the exclusion of crystals and the loss of film transparency (see above). Moreover, the higher solubility of amorphous trehalose increases its release rates from the films as compared to the release of a crystalline form. Last but not least, exothermic heat of the dissolution of amorphous trehalose, conversely to the endothermic dissolution of trehalose dihydrate, has been considered interesting for the production of foodstuffs and medicaments with a warming effect in the mouth [47]. On the other hand, it was proposed that a partial crystallization of trehalose into the dihydrate form supports its biostabilizing effect, because it reduces its devitrification by an increase in the glass-transition temperature [49]. In the “open” conformation that occurs in the dihydrate form but not in the trehalose glass (amorphous solid trehalose), all the hydroxygroups in trehalose participate in hydrogen-bonding, which enables them to mimic an aqueous environment in dehydrated biological materials [46].

### 3.6. Antimicrobial Activity

**Figure 11:**
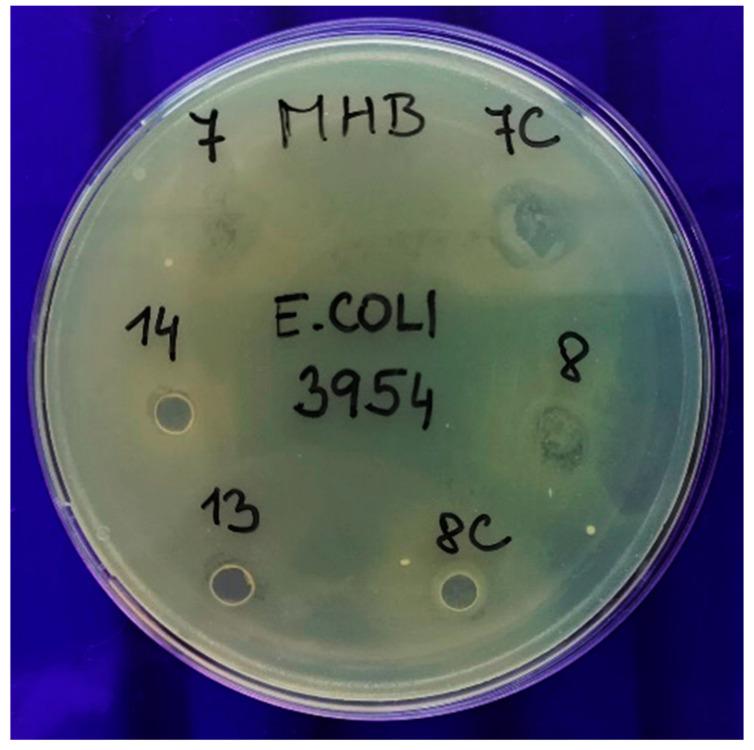
Evaluation of antimicrobial activity based on overgrowth of microbial culture over the disc of edible envelope; evaluation scale 1–5 (1 = disc completely clean; 5 = disc completely overgrown). The results are designated as follows: 7: 0.3gTr1OT80; 7C: 0.3gTr1; 8: 0.3gTr3OT80; 8C: 0.3gTr3; 13: OT20; 14: Tr0.5OT20.

The plates with edible films and bacteria after 18 h incubation were photographed and evaluated with respect to the evaluation scale mentioned earlier. The results are shown in Table 4. The Figure 11 describes one of the plates in more detail, explaining the evaluation. Clearly, in samples 7, 7C and 8, the bacteria have overgrown the edible film and the blue background is barely visible, and thus, these samples were evaluated as value 5, 3 and 4, respectively. On the other hand, the blue background is visible in samples 8C, 13 and 14, indicating no bacterial growth at all (value is 1 for all). The complete results are shown in Figure S7.

Based on the previous results, we divided the prepared edible films into three groups. The first group consisted of samples with the addition of 0.5 g of κ-carrageenan, and these samples were resistant to all three tested microorganisms. The second group consisted of films with the addition of 0.3 g κ-carrageenan and without trehalose, which were, similarly to the previous group, resistant to all three tested microorganisms. The third group consisted of films with the addition of 0.3 g κ-carrageenan and trehalose, and these samples were not resistant to *Candida albicans* and in some cases also to *Escherichia coli*. 

Most prepared edible films were resistant to *Staphylococcus aureus* subsp. *aureus* (MRSA), except for 0.3gTr3OT80. Generally, for the edible film to be active against Gram-negative bacteria (*Escherichia coli* CCM 3954) and the yeast *Candida albicans* CCM 8261, the necessary amount of κ-carrageenan was 0.5 g, with an exception for sample Tr3. The edible film prepared from 0.3 g of κ-carrageenan showed antimicrobial activity by itself or in combination with OEO and Tween 80. For activity against *E. coli*, combinations 0.3gOT20, 0.3gTr0.5, 0.3gTr3 and 0.3g κ 0.3gTr0.5OT80 were also critical. The results showed that with an increasing amount of trehalose in films, neither 0.3 g of κ-carrageenan nor OEO were able to inhibit growth of *E. coli* or *C. albicans*. Thus, we suggest that trehalose serves as a nutrient in these cases [50].

To summarize all the results of antimicrobial activity, prepared edible films were generally resistant to a Gram-positive bacterium, but in the case of Gram-negative bacteria or a yeast, only when 0.5 g of κ-carrageenan was used. We thus proved the usage of κ-carrageenan for the preparation of edible coatings to improve stability against microbial contamination.

### 3.7. Principal Component Analysis

**Figure 12:**
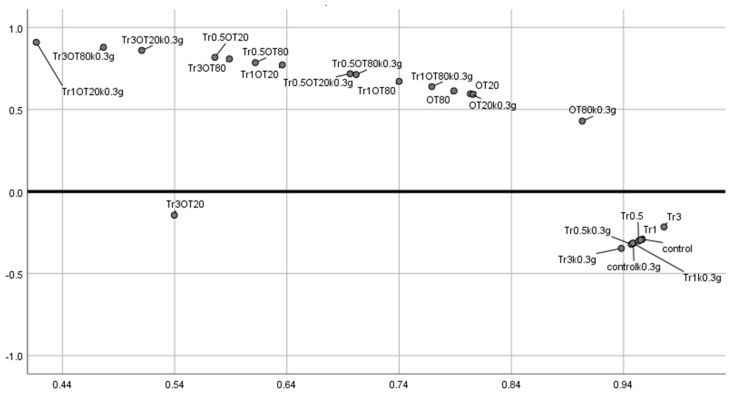
Overall PCA analysis.

The overall principal component analysis (PCA) is shown in Figure 12. According to the results obtained by PCA, one group consisting of control, Tr0.5, Tr1, Tr3 and control 0.3g, Tr0.5k0.3g, Tr1k0.3g, Tr3k0.3g can be seen. PCA also emphasized that the addition of orange essential oil, Tween 80 and Tween 20 had an impact on UV-Vis properties of prepared films. Another finding was that the addition of Tween 20 and Tween 80 did not affect the film matrix.

## 4. Conclusions

The research showed that the combination of trehalose with orange essential oil had a great impact on barrier protection against UV-VIS radiation; the protection was significantly higher than using orange essential oil (OEO) or trehalose alone. A synergistic effect was found between these two substances and potentially the usage of films for food packaging as protectants and improvers of shelf life and food quality. The most protective UV properties were found for samples with the highest concentrations of trehalose and essential oils (since the lowest transmittance values at 356 nm were observed). Furthermore, it was found that experimentally produced packaging showed the greatest antimicrobial activity against *Staphyloccocus aureus*. The highest antimicrobial activity against all studied microorganisms was shown by the edible films with the higher addition of carrageenan (0.5 g) in the film matrix. Using FTIR and SEM analysis, it was found that trehalose formation is dependent on the amount of carrageenan, the presence of orange essential oil and Tween addition. The compatibility of trehalose in the film matrix was better in the films with 0.3 g of carrageenan than with 0.5 g of carrageenan. The obtained results represent the knowledge improvement in properties of edible packaging prepared with trehalose, orange essential oil and carrageenan. Certainly, the findings are valuable for further research and possible application of edible packaging.

## Figures and Tables

**Table 1:** Composition of film samples.

Sample	Composition
c	0.5 g κ-carrageenan + glycerol
OT80	0.5 g κ-carrageenan + tween 80 + orange essential oil + glycerol
OT20	0.5 g κ-carrageenan + tween 20 + orange essential oil + glycerol
Tr0.5	0.5 g κ-carrageenan + 0.5% trehalose + glycerol
Tr0.5OT80	0.5 g κ-carrageenan + 0.5% trehalose + tween 80 + orange essential oil + glycerol
Tr0.5OT20	0.5 g κ-carrageenan + 0.5% trehalose + tween 20 + orange essential oil + glycerol
Tr1	0.5 g κ-carrageenan + 1% trehalose + glycerol
Tr1OT80	0.5 g κ-carrageenan + 1% trehalose + tween 80 + orange essential oil + glycerol
Tr1OT20	0.5 g κ-carrageenan + 1% trehalose + tween 20 + orange essential oil + glycerol
Tr3	0.5 g κ-carrageenan + 3% trehalose + glycerol
Tr3OT80	0.5 g κ-carrageenan + 3% trehalose + tween 80 + orange essential oil + glycerol
Tr3OT20	0.5 g κ-carrageenan + 3% trehalose + tween 20 + orange essential oil + glycerol
0.3g c	0.3 g κ-carrageenan + glycerol
0.3gOT80	0.3 g κ-carrageenan + tween 80 + orange essential oil + glycerol
0.3gOT20	0.3 g κ-carrageenan + tween 20 + orange essential oil + glycerol
0.3gTr0.5	0.3 g κ-carrageenan + 0.5% trehalose + glycerol
0.3gTr0.5OT80	0.3 g κ-carrageenan + 0.5% trehalose + tween 80 + orange essential oil + glycerol
0.3gTr0.5OT20	0.3 g κ-carrageenan + 0.5% trehalose + tween 20 + orange essential oil + glycerol
0.3gTr1	0.3 g κ-carrageenan + 1% trehalose + glycerol
0.3gTr1OT80	0.3 g κ-carrageenan + 1% trehalose + tween 80 + orange essential oil + glycerol
0.3gTr1OT20	0.3 g κ-carrageenan + 1% trehalose + tween 20 + orange essential oil + glycerol
0.3gTr3	0.3 g κ-carrageenan + 3% trehalose + glycerol
0.3gTr3OT80	0.3 g κ-carrageenan + 3% trehalose + tween 80 + orange essential oil + glycerol
0.3gTr3OT20	0.3 g κ-carrageenan + 3% trehalose + tween 20 + orange essential oil + glycerol

**Table 2:** Transmittance of films.

Sample	T_356_ (%)	T_600_ (%)	Sample	T_356_ (%)	T_600_ (%)
**c**	82.20 ± 0.02 ^a^	88.43 ± 0.03 ^a^	**0.3g c**	83.79 ± 0.05 ^a^	87.69 ± 0.02 ^a^
**OT80**	7.07 ± 1.22 ^bdfgh^	42.06 ± 1.15 ^b^	**0.3gOT80**	28.23 ± 0.13 ^b^	87.81 ± 0.35 ^ac^
**OT20**	10.81 ± 0.04 ^d^	84.31 ± 0.03 ^c^	**0.3gOT20**	11.77 ± 0.01 ^c^	82.14 ± 0.04 ^b^
**Tr0.5**	75.52 ± 0.06 ^c^	80.76 ± 0.08 ^d^	**0.3gTr0.5**	83.63 ± 0.06 ^a^	87.30 ± 0.02 ^c^
**Tr0.5OT80**	1.02 ± 0.13 ^bg^	14.56 ± 1.16 ^eghi^	**0.3gTr0.5OT80**	5.55 ± 0.03 ^d^	21.85 ± 0.03 ^d^
**Tr0.5OT20**	1.54 ± 0.03 ^b^	8.50 ± 0.02 ^e^	**0.3gTr0.5OT20**	4.42 ± 0.15 ^e^	21.23 ± 0.27 ^d^
**Tr1**	75.06 ± 0.07 ^e^	81.03 ± 0.04 ^d^	**0.3gTr1**	80.69 ± 2.68 ^af^	85.12 ± 2.21 ^abc^
**Tr1OT80**	3.52 ± 0.73 ^bfgh^	21.37 ± 0.27 ^f^	**0.3gTr1OT80**	6.33 ± 0.05 ^g^	22.05 ± 0.07 ^d^
**Tr1OT20**	1.88 ± 0.01 ^f^	10.94 ± 0.01 ^g^	**0.3gTr1OT20**	1.23 ± 0.01 ^h^	9.09 ± 0.04 ^e^
**Tr3**	64.14 ± 2.21 ^ce^	76.06 ± 1.37 ^d^	**0.3gTr3**	67.61 ± 0.14 ^f^	69.51 ± 0.03 ^f^
**Tr3OT80**	0.17 ± 0.05 ^h^	9.00 ± 0.02 ^h^	**0.3gTr3OT80**	0.61 ± 0.01 ^i^	3.05 ± 0.01 ^g^
**Tr3OT20**	0.69 ± 0.00 ^g^	9.79 ± 0.03 ^i^	**0.3gTr3OT20**	0.14 ± 0.02 ^j^	1.85 ± 0.00 ^h^

Letters in superscript indicate statistically significant (*p* < 0.05) differences between rows.

**Table 3:** Transparency value of films.

Sample	Transparency Value	Sample	Transparency Value
**c**	23.36 ± 0.00 ^a^	**0.3g c**	29.89 ± 0.00 ^a^
**OT80**	6.86 ± 0.05 ^b^	**0.3gOT80**	10.41 ± 0.01 ^b^
**OT20**	8.08 ± 0.00 ^c^	**0.3gOT20**	9.49 ± 0.00 ^c^
**Tr0.5**	18.16 ± 0.00 ^d^	**0.3gTr0.5**	27.08 ± 0.00 ^d^
**Tr0.5OT80**	4.41 ± 0.13 ^ei^	**0.3gTr0.5OT80**	7.80 ± 0.00 ^e^
**Tr0.5OT20**	3.60 ± 0.00 ^f^	**0.3gTr0.5OT20**	6.86 ± 0.03 ^f^
**Tr1**	15.07 ± 0.00 ^g^	**0.3gTr1**	21.44 ± 0.12 ^g^
**Tr1OT80**	4.93 ± 0.02 ^e^	**0.3gTr1OT80**	6.83 ± 0.01 ^f^
**Tr1OT20**	3.92 ± 0.00 ^i^	**0.3gTr1OT20**	4.83 ± 0.01 ^h^
**Tr3**	8.12 ± 0.03 ^c^	**0.3gTr3**	9.96 ± 0.00 ^i^
**Tr3OT80**	2.65 ± 0.00 ^h^	**0.3gTr3OT80**	1.59 ± 0.00 ^j^
**Tr3OT20**	2.61 ± 0.00 ^j^	**0.3gTr3OT20**	0.77 ± 0.00 ^k^

Letters in superscript indicate statistically significant (*p* < 0.05) differences between rows.

**Table 4:** Evaluation of antimicrobial activity based on overgrowth of microbial culture over the disc of edible films; evaluation scale 1–5 (1 = disc completely clean, whereas 5 = disc completely overgrown); therefore, + means antimicrobial activity (1,2) and – means no activity (3, 4, 5).

Sample	No.	Antimicrobial activity against *Staphylococcus aureus* subsp. *aureus* (MRSA) CCM 7110	Antimicrobial activity against *Escherichia coli* CCM 3954	Antimicrobial activity against *Candida albicans* CCM 8261
c	1C	+ (2)	+ (1)	+ (2)
OT80	1	+ (2)	+ (1)	+ (1)
OT20	13	+ (1)	+ (1)	+ (2)
Tr0.5	2C	+ (2)	+ (1)	+ (2)
Tr0.5OT80	2	+ (1)	+ (1)	+ (2)
Tr0.5OT20	14	+ (1)	+ (1)	+ (2)
Tr1	3C	+ (1)	+ (1)	+ (1)
Tr1OT80	3	+ (1)	+ (1)	+ (1)
Tr1OT20	15	+ (1)	+ (2)	+ (2)
Tr3	4C	+ (2)	+ (1)	− (4)
Tr3OT80	4	+ (2)	+ (1)	+ (2)
Tr3OT20	16	+ (2)	+ (1)	+ (2)
0.3g c	5C	+ (1)	+ (2)	+ (2)
0.3gOT80	5	+ (1)	+ (1)	+ (2)
0.3gOT20	17	+ (2)	+ (2)	− (5)
0.3gTr0.5	6C	+ (1)	+ (1)	− (5)
0.3gTr0.5OT80	6	+ (1)	+ (1)	− (3)
0.3gTr0.5OT20	18	+ (2)	− (3)	− (3)
0.3gTr1	7C	+ (2)	− (3)	− (3)
0.3gTr1OT80	7	+ (2)	− (5)	− (3)
0.3gTr1OT20	19	+ (2)	− (5)	− (3)
0.3gTr3	8C	+ (2)	+ (1)	− (3)
0.3gTr3OT80	8	− (4)	− (4)	− (4)
0.3gTr3OT20	20	+ (2)	− (5)	− (3)

## Supplementary Materials

The following are available online at https://www.mdpi.com/2073-4360/13/3/332/s1, Figure S1: The appearance of films that consisted of 0.5 g of κ-carrageenan; Figure S2: The appearance of films that consisted of 0.3 g of κ-carrageenan; Figure S3: ATR FTIR spectra of the trehalose-containing carrageenan films with the addition of essential oil (0.5 g of carrageenan, Tween 20); Figure S4: ATR FTIR spectra of the trehalose-containing carrageenan films without addition of essential oil (0.3 g of carrageenan); Figure S5: ATR FTIR spectra of the trehalose-containing carrageenan films with the addition of essential oil (0.3 g of carrageenan, Tween 80); Figure S6: ATR FTIR spectra of the trehalose-containing carrageenan films with the addition of essential oil (0.3 g of carrageenan, Tween 20); Figure S7: The complete results of antimicrobial activity of prepared films.

## References

[1] Lee D.S., Yam K.L., Piergiovanni L. (2008). Food Packaging Science and Technology.

[2] Ramos Ó.L., Reinas I., Silva S.I., Fernandes J.C., Cerqueira M.A., Pereira R.N., Vicente A.A., Poças M.F., Pintado M.E., Malcata F.X. (2013). Effect of whey protein purity and glycerol content upon physical properties of edible films manufactured therefrom. Food Hydrocoll..

[3] Ramos M., Valdes A., Beltran A., Garrigós M.C. (2016). Gelatin-based films and coatings for food packaging applications. Coatings.

[4] Wang Z., Tang L., Lin F., Shen Y., Chen Y., Chen X., Huang B., Lu B. (2020). Multi-Functional Edible Film with Excellent UV Barrier Performance and Accurate Instant Ion Printing Capability. Adv. Sustain. Syst..

[5] Farajpour R., Djomeh Z.E., Moeini S., Tavahkolipour H., Safayan S. (2020). Structural and physico-mechanical properties of po-tato starch-olive oil edible films reinforced with zein nanoparticles. Int. J. Biol. Macromol..

[6] Bourtoom T. (2008). Edible films and coatings: Characteristics and properties. Int. Food Res. J..

[7] Albanese D., Cinquanta L., Di Matteo M. (2007). Effects of an innovative dipping treatment on the cold storage of minimally processed Annurca apples. Food Chem..

[8] Yildiz F., Wiley R.C. (2017). Minimally Processed Refrigerated Fruits and Vegetables.

[9] Ayhan Z. (2017). Packaging and Preservation Methods of Minimally Processed Produce. Agents of Change.

[10] Richards A., Krakowka S., Dexter L., Schmid H., Wolterbeek A., Waalkens-Berendsen D., Shigoyuki A., Kurimoto M. (2002). Trehalose: A review of properties, history of use and human tolerance, and results of multiple safety studies. Food Chem. Toxicol..

[11] Liu T., Zhu L., Zhang Z., Huang H., Zhang Z., Jiang L. (2017). Protective role of trehalose during radiation and heavy metal stress in *Aureobasidium subglaciale* F134. Sci. Rep..

[12] Lv F., Liang H., Yuan Q., Li C. (2011). In vitro antimicrobial effects and mechanism of action of selected plant essential oil com-binations against four food-related microorganisms. Food Res. Int..

[13] Maisanaba S., Llana-Ruiz-Cabello M., Gutiérrez-Praena D., Pichardo S., Puerto M., Prieto A.I., Cameán A.M. (2017). New ad-vances in active packaging incorporated with essential oils or their main components for food preservation. Food Rev. Int..

[14] Pelissari F.M., Grossmann M.V., Yamashita F., Pineda E.A.G. (2009). Antimicrobial, mechanical, and barrier properties of cas-sava starch− chitosan films incorporated with oregano essential oil. J. Agric. Food Chem..

[15] Sánchez-González L., Vargas M., González-Martínez C., Chiralt A., Cháfer M. (2011). Use of Essential Oils in Bioactive Edible Coatings: A Review. Food Eng. Rev..

[16] Kaur C.D., Saraf S. (2010). In vitro sun protection factor determination of herbal oils used in cosmetics. Pharmacogn. Res..

[17] Frassinetti S., Caltavuturo L., Cini M., Della Croce C.M., Maserti B.E. (2011). Antibacterial and antioxidant activity of essential oils from Citrus spp.. J. Essent. Oil Res..

[18] Sabo B., Bečica T., Keleš N., Kovačević D., Brozović M. (2017). The impact of packaging transparency on product attractiveness. J. Graph. Eng. Des..

[19] Cagri A., Ustunol Z., Ryser E.T. (2004). Antimicrobial Edible Films and Coatings. J. Food Prot..

[20] Escamilla-García M., Calderón-Domínguez G., Chanona-Pérez J.J., Mendoza-Madrigal A.G., Di Pierro P., García-Almendárez B.E., Amaro-Reyes A., Regalado-González C. (2017). Physical, Structural, Barrier, and Antifungal Characterization of Chitosan–Zein Edible Films with Added Essential Oils. Int. J. Mol. Sci..

[21] Siracusa V., Romani S., Gigli M., Mannozzi C., Cecchini J.P., Tylewicz U., Lotti N. (2018). Characterization of Active Edible Films based on Citral Essential Oil, Alginate and Pectin. Materials.

[22] Du W.X., Avena-Bustillos R.J., Hua S.S.T., McHugh T.H. (2011). Antimicrobial volatile essential oils in edible films for food safety. Sci. Against Microb. Pathog. Commun. Curr. Res. Technol. Adv..

[23] Han J.H., Floros J.D. (1997). Casting Antimicrobial Packaging Films and Measuring Their Physical Properties and Antimicrobial Activity. J. Plast. Film Sheeting.

[24] Shankar S., Rhim J.W. (2017). Preparation and characterization of agar/lignin/silver nanoparticles composite films with ultravio-let light barrier and antibacterial properties. Food Hydrocoll..

[25] Ahmed J., Arfat Y.A., Al-Attar H., Auras R., Ejaz M. (2017). Rheological, structural, ultraviolet protection and oxygen barrier properties of linear low- density polyethylene films reinforced with zinc oxide (ZnO) nanoparticles. Food Packag. Shelf Life.

[26] Jancikova S., Dordevic D., Jamroz E., Behalova H., Tremlova B. (2020). Chemical and Physical Characteristics of Edible Films, Based on κ- and ι-Carrageenans with the Addition of Lapacho Tea Extract. Foods.

[27] Wu J., Sun X., Guo X., Ge S., Zhang Q. (2017). Physicochemical properties, antimicrobial activity and oil release of fish gelatin films incorporated with cinnamon essential oil. Aquac. Fish..

[28] Ploypetchara T., Gohtani S. (2020). Characteristics of rice starch film blended with sugar (trehalose/allose) and oil (canola oil/coconut oil): Part I—Filmogenic solution behavior and mechanical properties. J. Food Sci..

[29] Haghighi H., Biard S., Bigi F., De Leo R., Bedin E., Pfeifer F., Siesler H.W., Licciardello F., Pulvirenti A. (2019). Comprehensive characterization of active chitosan-gelatin blend films enriched with different essential oils. Food Hydrocoll..

[30] Liu Y., Qin Y., Bai R., Zhang X., Yuan L., Liu J. (2019). Preparation of pH-sensitive and antioxidant packaging films based on κ-carrageenan and mulberry polyphenolic extract. Int. J. Biol. Macromol..

[31] Zavala J.A., Ravetta D.A. (2002). The effect of solar UV-B radiation on terpenes and biomass production in Grindelia chiloensis (Asteraceae), a woody perennial of Patagonia, Argentina. Plant. Ecol..

[32] Bekbölet M. (1990). Light Effects on Food. J. Food Prot..

[33] Forney L.J., Moraru C.I. (2009). Ultraviolet Light in Food Technology: Principles and Applications.

[34] El-Tinay A.H., Chichester C.O. (1970). Oxidation of beta-carotene. Site of initial attack. J. Org. Chem..

[35] Rincón E., Serrano L., Balu A.M., Aguilar J.J., Luque R., García A. (2019). Balu Effect of Bay Leaves Essential Oil Concentration on the Properties of Biodegradable Carboxymethyl Cellulose-Based Edible Films. Materials.

[36] Nowicki M., Richter A., Wolf B., Kaczmarek H. (2003). Nanoscale mechanical properties of polymers irradiated by UV. Polymer.

[37] Diepens M., Gijsman P. (2007). Photodegradation of bisphenol A polycarbonate. Polym. Degrad. Stab..

[38] Yousif E., Haddad R. (2013). Photodegradation and photostabilization of polymers, especially polystyrene: Review. SpringerPlus.

[39] Nor M.H.M., Nazmi N.N.M., Sarbon N.M. (2017). Effects of plasticizer concentrations on functional properties of chicken skin gelatin films. Int. Food Res. J..

[40] Šuput D., Lazić V., Pezo L., Markov S., Vaštag Ž., Popović L., Radulović A., Ostojić S., Zlatanović S., Popović S. (2016). Charac-terization of starch edible films with different essential oils addition. Pol. J. Food Nutr. Sci..

[41] Shojaee-Aliabadi S., Hosseini H., Mohammadifar M.A., Mohammadi A., Ghasemlou M., Ojagh S.M., Hosseini S.M., Khaksar R. (2013). Characterization of antioxidant-antimicrobial κ-carrageenan films containing Satureja hortensis essential oil. Int. J. Biol. Macromol..

[42] Gómez-Guillén M.D.C., Ihl M., Bifani V., Silva A., Montero P. (2007). Edible films made from tuna-fish gelatin with antioxidant extracts of two different murta ecotypes leaves (Ugni molinae Turcz). Food Hydrocoll..

[43] Edhirej A., Sapuan S.M., Jawaid M., Zahari N.I. (2016). Effect of various plasticizers and concentration on the physical, thermal, mechanical, and structural properties of cassava-starch-based films. Starch Stärke.

[44] Ortiz-Tafoya M., Tecante A. (2018). Physicochemical characterization of sodium stearoyl lactylate (SSL), polyoxyethylene sorbitan monolaurate (Tween 20) and κ-carrageenan. Data Brief..

[45] Sussich F., Urbani R., Princivalle F., Cesàro A. (1998). Polymorphic Amorphous and Crystalline Forms of Trehalose. J. Am. Chem. Soc..

[46] Akao K.-I., Okubo Y., Asakawa N., Inoue Y., Sakurai M. (2001). Infrared spectroscopic study on the properties of the anhydrous form II of trehalose. Implications for the functional mechanism of trehalose as a biostabilizer. Carbohydr. Res..

[47] Cooper J.M., Tian W.U.S. (2003). Edible Compositions Containing Trehalose. U.S. Patent.

[48] Pérez L., Piccirilli G., Delorenzi N.J., Verdini R. (2016). Effect of different combinations of glycerol and/or trehalose on physical and structural properties of whey protein concentrate-based edible films. Food Hydrocoll..

[49] Aldous B.J., Auffret A.D., Franks F. (1995). The crystallisation of hydrates from amorphous carbohydrates). Cryoletters.

[50] Elbein A.D., Pan Y., Pastuszak I., Carroll D. (2003). New insights on trehalose: A multifunctional molecule. Glycobiology.

