# Supplementary material for: Edible Films from Carrageenan/Orange Essential Oil/Trehalose—Structure, Optical Properties, and Antimicrobial Activity
Source: Polymers (Basel). 2021 Jan 21;13(3):332. doi: 10.3390/polym13030332 (PMC7864528; doi:10.3390/polym13030332)
Supplement: Supplementary file 1 [file polymers-13-00332-s001.pdf]

polymers

MDPI

Supplementary material

Edible films from carrageenan/orange essential oil/trehalose – structure, optical properties, and antimicrobial activity

Jancikova Simona [1*], Dordevic Dani[1], Sedlacek Petr[2], Nejezchlebova Marcela[3], Treml Jakub[3] and Tremlova Bohuslava[1]

[1] Department of Vegetable Foodstuffs Hygiene and Technology, Faculty of Veterinary Hygiene and Ecology, University of Veterinary and Pharmaceutical Sciences Brno, Palackeho tr. 1946/1, 612 42 Brno, Czech Republic; jancikovas@vfu.cz (S.J.); dordevicd@vfu.cz (D.D.); tremlovab@vfu.cz (B.T.)

[2] Faculty of Chemistry, Brno University of Technology, Purkynova 118, 612 00 Brno, Czech Republic

[3] Department of Molecular Pharmacy, Faculty of Pharmacy, Masaryk University, Palackeho tr. 1946/1, 612 00 Brno, Czech Republic

* Correspondence: jancikovas@vfu.cz; Tel.: +420 774 607 936

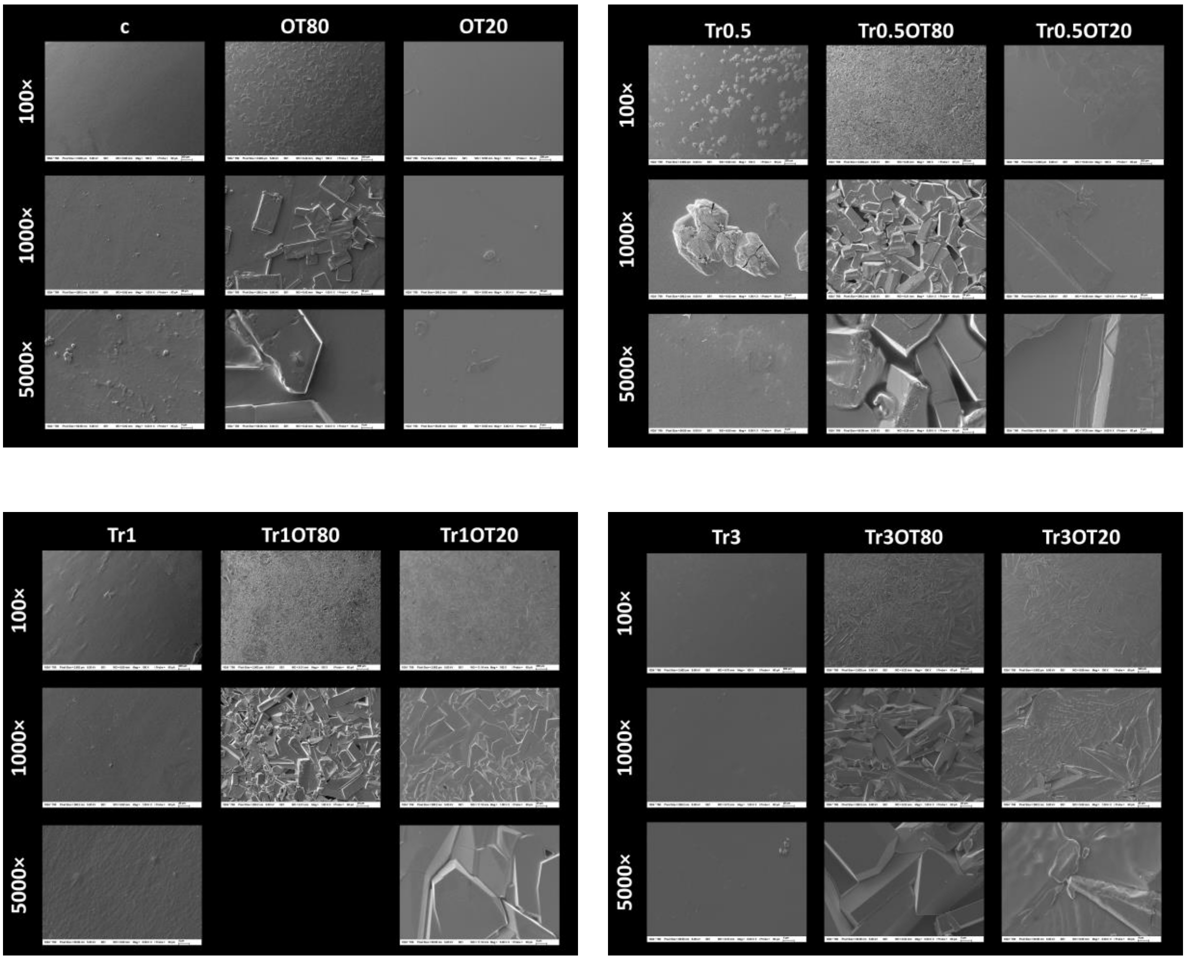

Figure S1:.The appearance of films consisted of 0.5 g of κ-carrageenan

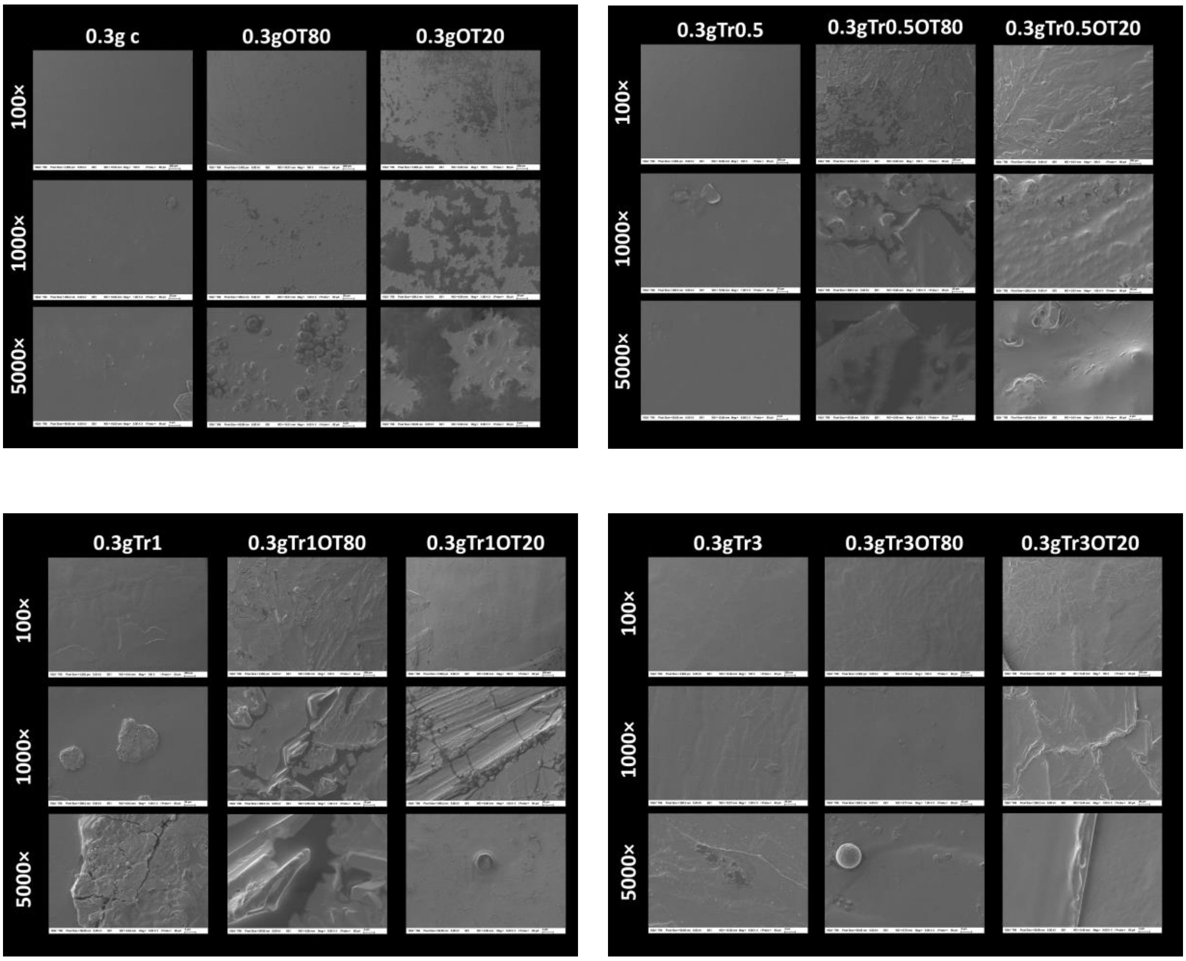

Figure S2. The appearance of films consisted of 0.3 g of κ-carrageenan

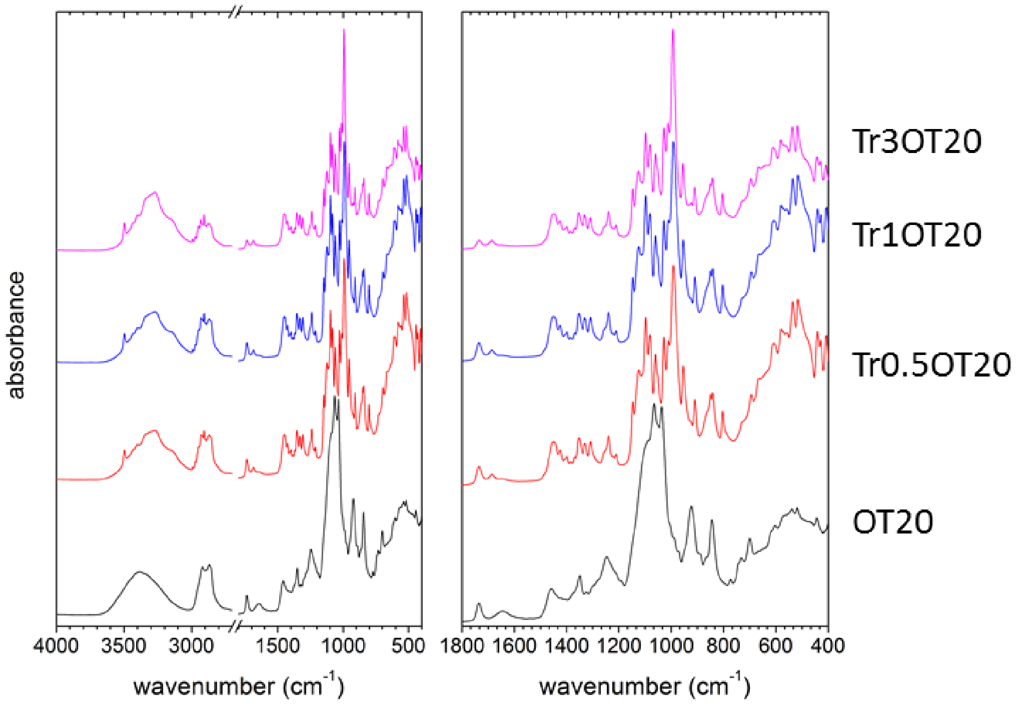

Figure S3. ATR FTIR spectra of the trehalose-containing carrageenan films with the addition of essential oil (0.5 g of carrageenan, Tween 20)

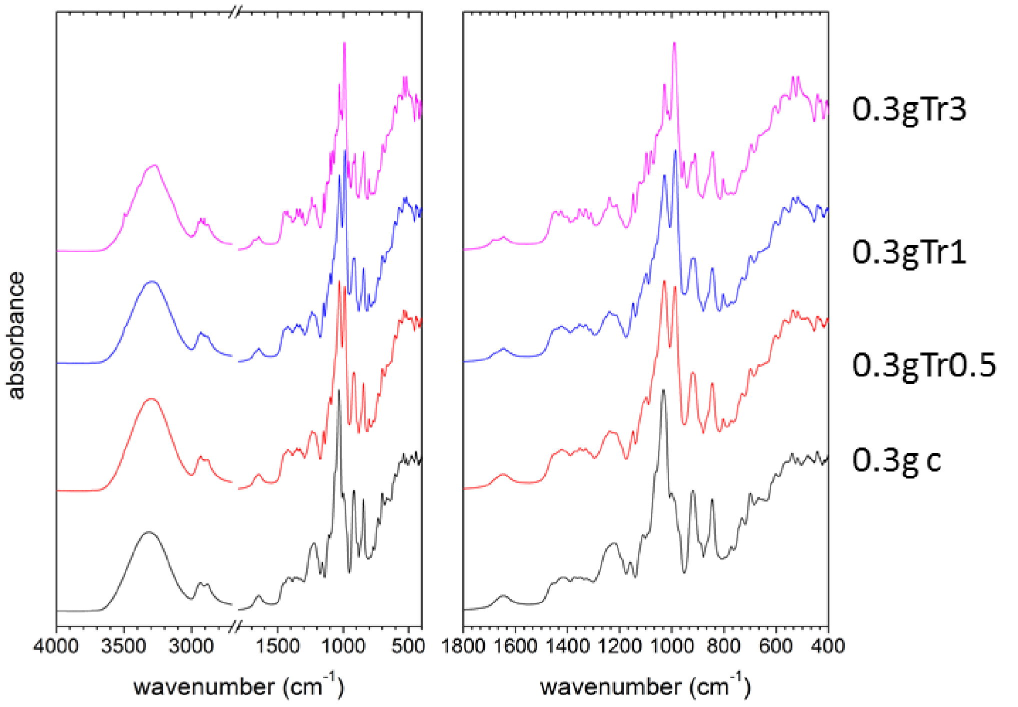

Figure S4. ATR FTIR spectra of the trehalose-containing carrageenan films without addition of essential oil (0.3 g of carrageenan)

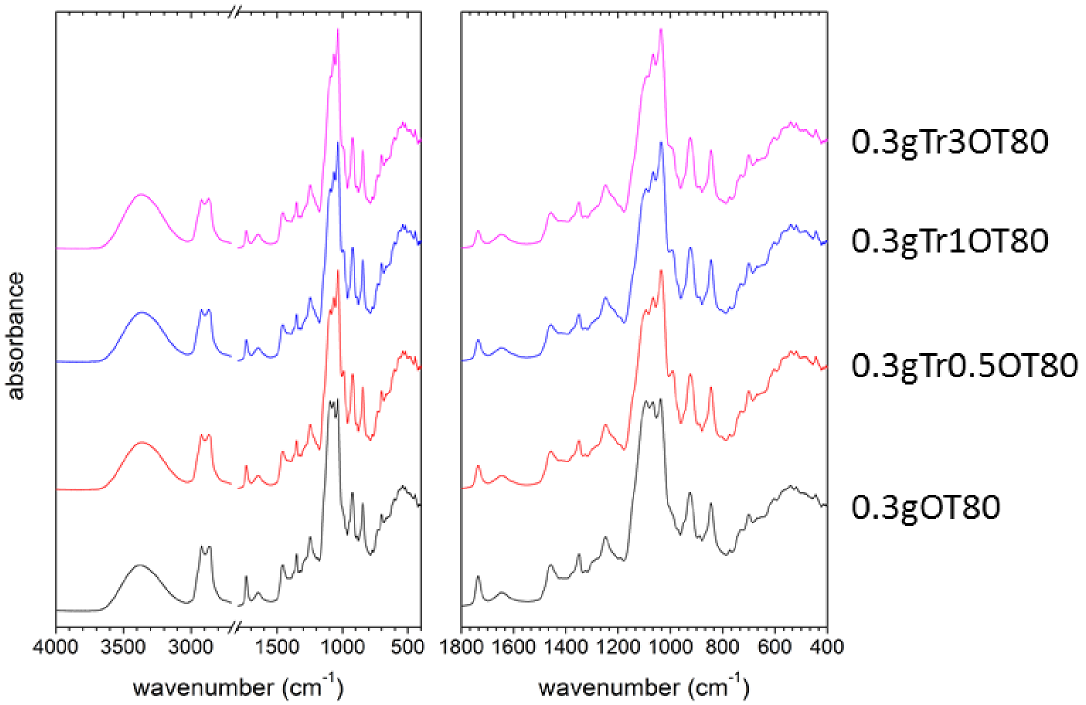

Figure S5. ATR FTIR spectra of the trehalose-containing carrageenan films with the addition of essential oil (0.3 g of carrageenan, Tween 80)

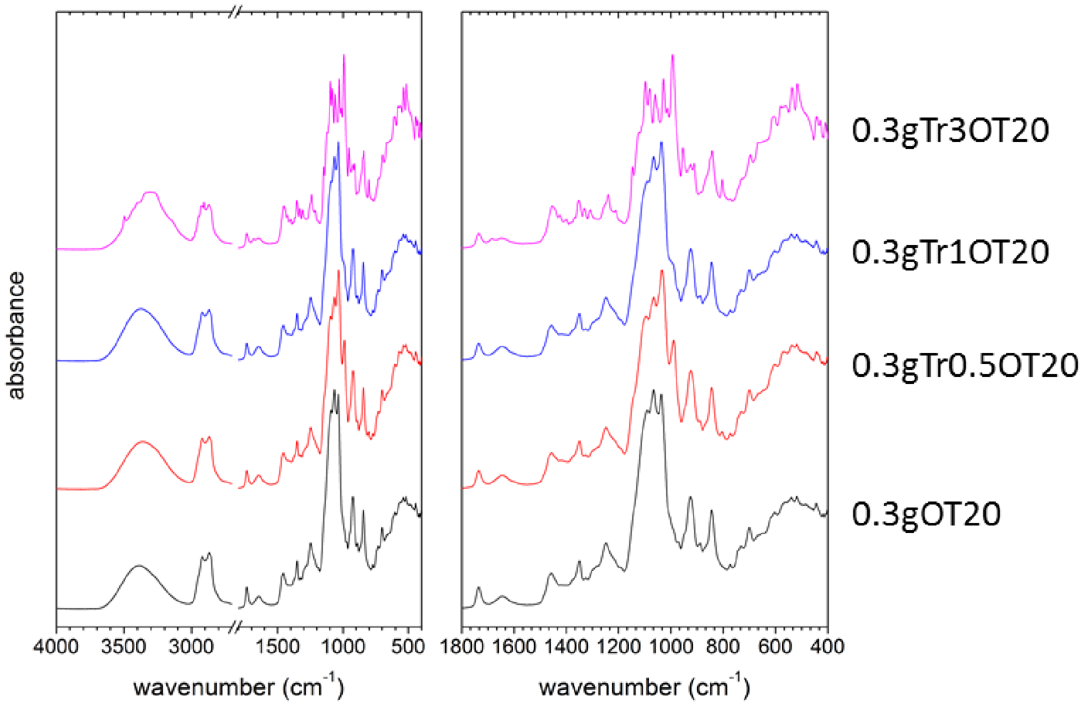

Figure S6. ATR FTIR spectra of the trehalose-containing carrageenan films with the addition of essential oil (0.3 g of carrageenan, Tween 20)

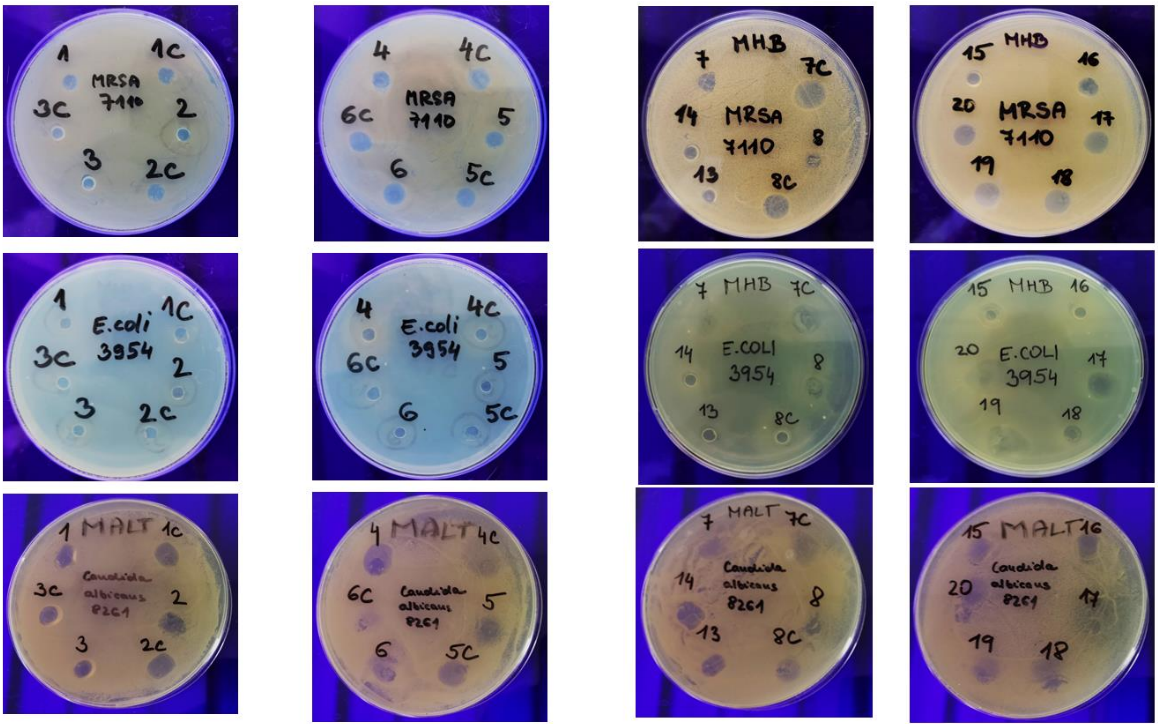

Figure S7. The complete results of antimicrobial activity of prepared films
